# Team Cohesion in Individual/Team Sports Athletes: Transformational Leadership and the Role of Social norms

**DOI:** 10.3390/healthcare11060792

**Published:** 2023-03-08

**Authors:** Youngtaek Oh, Jung-In Yoo

**Affiliations:** 1 Department of Kinesiology & Sport Management, Texas A&M University, College Station, TX 77843, USA; 2 Division of Sports Science, College of Health Science, The University of Suwon, Hwaseong 18323, Republic of Korea

**Keywords:** transformational leadership, social norm, team cohesion, sport culture, individual sports and team sports

## Abstract

Team cohesion is a critical factor in sports, yet few studies have comparatively analyzed individual and team sport athletes in sporting situations. The purpose of this study was to identify the relationship between transformational leadership, social norms, and team cohesion, and to analyze the moderating effects of individual/team sports athletes. In 2022, a total of 196 baseball, judo, soccer, taekwondo, and hockey players registered with the Korean Sport & Olympic Committee completed a questionnaire using transformational leadership, social norms, and team cohesion scales. Transformational leadership had a significant positive effect on social norms and team cohesion. Social norms had a significant positive effect on team cohesion. Transformational leadership and the interaction of individual/team sports athletes had a significant effect on team cohesion. At this time, individual sports athletes appeared to have somewhat higher team cohesion. This study sheds light on the social norms and team cohesion of athletes from a social moral perspective based on transformational leadership theory. It can also help young athletes who are just starting out to learn the culture and sociology of sports.

## 1. Introduction

Team sports athletes systematically organize team tactics and strategies among their team members to derive the best performance [1]. In the study of sports, team cohesion is mainly studied in team sports athletes [2]. The study considered whether team cohesion, which is important for team athletes, would also be important for individual athletes. The study was designed with this question in mind. As transformational leadership and individual/team sports athletes are significantly related, it is important to have a detailed understanding of the inner workings of transformational leadership and the processes underlying its development [3,4].

This study examined the relationship between transformational leadership, social norms, and team cohesion, as perceived by individual and team sports athletes. Team cohesion refers to ‘the force that acts on all members of a group to keep them working within a group’ [5]. Carron et al. [6] described this concept as a dynamic process dealing with the tendency to collectively converge for an active purpose and the satisfaction of peer emotional needs. The terms “team cohesion” and “teamwork” are sometimes used interchangeably [7]. Team cohesion was closely related to self-efficacy, emotional intelligence [8], and communication [1,9]. MacKinnon et al. [10] emphasized efforts to identify parameters that can convey these effects when relationships between independent and dependent variables are found. Therefore, in this study, the transformational leadership of the leader, which is closely related to the athletes, was assumed as an independent variable.

Transformational leadership theory has been a major topic of interest for many researchers in the field of organizational leadership in the past [11]. It was first developed by Bums [12] and later improved by Bass [13,14] and other scholars [15,16,17]. The main quality of transformational leadership is the leader’s ability to motivate members to achieve beyond their planned goals [18]. In the sports context, transformational leadership is widely applied to strengthen the relationship between coaching and athletes [13,19]. Several scholars have argued that coaches’ high-level transformational leadership is closely related to athletes’ performance [20,21], well-being [22], and team cohesion [23,24].

It is not widely understood how the relationship between transformational leadership and member outcomes in the sports domain is mediated [4]. Chan et al. [25] recently suggested that it is important to continuously investigate mechanisms that promote transformational leadership and team cohesion for organizational development. The mediators in our study are team social norms that can improve transformational leadership and team cohesion perceived by athletes. Social norms in a sports team refer to the common expectations for the behavior of its members, which determine what the group must do and how to maintain consistent and desirable behavior [1]. These norms are informal rules that evolve among team members, either as they evolve around the importance given to team rules or policies, or once determined, become formalized. However, these rules are not policies that must be followed in the same way by other teams of the same sport or by athletes in all sports [26]. Carron et al. [27] explained that norms play an important role in sports teams because they are essential for the development and functioning of team members and provide a sense of legitimacy and excellence.

Meanwhile, previous studies have investigated the relationship between the variables in sports such as soccer [28], basketball [29], volleyball [30], swimming, handball [31], kumdo, and taekwondo [32] for athletes. Therefore, the strength of this study lies in its verification of the influence on team cohesion by analyzing two distinct groups: athletes in individual and team sports. By analyzing individual and team sports athletes separately, this study aims to understand team culture and atmosphere and emphasize the leadership role of coaching. The transformational leadership of the team coaches (head coach or assistant coach) will be examined to verify the relationship between team cohesion and athlete performance. In addition, team norms that help create a positive team atmosphere will reduce harm to the team by promoting discipline between coaches and athletes and enhance team cohesion by considering teammates. The importance of team cohesion in satisfying members’ desire to belong to a team atmosphere and strengthening organizational power has been emphasized by Bruner et al. [33].

The goal of this study was to investigate whether there is a difference in the perception of transformational leadership by individual/team sports athletes based on the level of team cohesion. The study highlights the importance of social norms, team education, and team culture in enhancing team cohesion. Understanding how athletes can increase team cohesion is a crucial topic for researchers, sports socio-psychology managers, and coaches. Therefore, the study aimed to explore the relationship between transformational leadership, social norms, and team cohesion as perceived by individual/team sports athletes.

### 1.1. Hypothesis Development

#### 1.1.1. The Relationship between Transformational Leadership, Social norms, and Team Cohesion

Transformational leadership has been widely used in sports to improve athlete outcomes through coaching behavior [13,19,34]. This type of leadership involves members overcoming selfishness and recognizing their limitations to achieve collective goals [35]. To measure transformational leadership, various scales have been used, such as the Multifactor Leadership Questionnaire [36], the Conger et al. [37] scale, and the Leadership Practices Inventory [38]. However, Podsakoff et al. [39] suggested that short and practical sentences are more appropriate for assessing transformational leadership due to the length and time-consuming nature of these scales [40]. Specifically, the scale should identify the team’s vision, facilitate group goal acceptance, provide athletes with appropriate role models, high expectations, individualized support, and intellectual stimulation. In this study, a short and clearly structured questionnaire developed by Carless et al. [40] was used to confirm the effects of social norms and team cohesion.

An emerging consensus in the coaching literature is that coaches’ transformational leadership predicts athletes’ perceptions of cohesion [41,42]. Cohesion was defined by Festinger et al. [5] as “the total field of forces which act on members to keep them working in the group”. Carron et al. [6] described cohesion as the process through which groups integrate due to active goals and to satisfy peers’ emotional needs. “Team unity” and “team chemistry” are often used interchangeably with the term “cohesion,” which encompasses all major group variables [1,7]. Spink et al. [43] found that team cohesiveness is positively correlated with athletes’ satisfaction and leadership behavior. We assume that social norms can serve as mediators in the relationship between transformational leadership and team cohesion by emphasizing team history and culture.

Feldman [44] discussed research linking team norms to team social processes. The effect of team norms on team cohesion is based on the social interdependence theory [45,46]. Social norms can promote team effectiveness and team member bonding by ensuring that team members consistently work toward desired goals and behavioral standards and that the team is cohesive. We will investigate the relationship between the coach’s transformational leadership and team cohesion in this study. In doing so, we will establish a causal link between the utilization of the team social norms of coaches and athletes. Therefore, research Hypotheses 1–3 are as follows.

**Hypothesis** **1** **(H1).** *Transformational leadership will have a significant effect on team cohesion*.

**Hypothesis** **2** **(H2).** *Transformational leadership will have a significant effect on team social norms*.

**Hypothesis** **3** **(H3).** *Team social norms will have a significant effect on team cohesion*.

#### 1.1.2. Team Culture and Individual/Team Sports

Hill et al. [47] argued that team members should play an important role in determining behavioral norms because they are influenced by cultural value orientations. This study emphasizes that an individual’s internalization of a team’s expectations and values can influence their behavior. To examine the organizational dynamics among athletes from different sports, we categorized participants in martial arts and record events as individual sport athletes, while those in ball games were categorized as team sports athletes. Ball game athletes were categorized as team event athletes. The relationship between transformational leadership and team cohesion perceived by the athletes of the two groups were confirmed. The results highlight the team cohesion of players in a particular sport. If both groups interact significantly, it can be emphasized that team cohesion is vital in a sporting environment. The model can be developed as a preceding model to improve team culture and organizational power. Therefore, research Hypotheses 4 is as follows:

**Hypothesis** **4** **(H4).** *Athletes’ perceptions of transformational leadership have a significant effect on team cohesion in both individual and team sports events*.

## 2. Materials and Methods

### 2.1. Participants

This study consisted of university athletes’ who completed an athletes’ registration at the 2022 Korean Sport and Olympic Committee. A total of 196 athletes were selected for the study, 151 (77.0%) of whom were male and 45 (23.0%) of whom were female. The sports these athletes participated in included baseball (34, 17.3%), judo (30, 15.3%), soccer (37, 18.9%), taekwondo (86, 43.9%), and hockey (9, 4.6%). Regarding player experience, there were players (13, 6.6%) with 1 to 5 years of experience, players with 5 to 10 years (128, 65.3%), and players with more than 10 years (55, 28.1%). 

### 2.2. Procedure

#### Questionnaire Scales

The questionnaire comprised scales of reliability and validity which had been adequately assessed in previous studies. We adopted seven items from Carless et al. [40] to measure transformational leadership, including ‘Encourages thinking about problems in new ways and questions assumptions’. These items were measured on a 5-point Likert-type scale (1 = strongly disagree; 5 = strongly agree). Confirmatory factor analysis revealed a relatively good fit index (ꭓ^2^ = 22.398, d*f* = 13, *p* < 0.01, Q = 1.723, IFI = 0.993, TLI = 0.998, CFI = 0.993, RMSEA = 0.061). We used 8 items from Munroe et al. [48] to measure social norms (Team Sports Competition Norm Questionnaire, TSCNQ). These included ‘Embarrass the group in a social situation’ and were measured on a 9-point Likert-type scale (1 = strongly disagree; 9 = strongly agree; reverse scoring). Confirmatory factor analysis revealed a relatively good fit index (ꭓ^2^ = 7.178, d*f* = 4, *p* < 0.001, Q = 1.795, IFI = 0.997, TLI = 0.992, CFI = 0.997, RMSEA = 0.064). Three items pertaining to team cohesion were adopted from Dion [49], including ‘Our team is united in trying to reach its goals for performance’, and they were measured on a 5-point Likert-type scale (1 = strongly disagree; 5 = strongly agree). While the RMSEA did not support a good model fit, the results of the confirmatory factor analysis (CFA) indicated a relatively good fit index (ꭓ^2^ = 8.174, d*f* = 1, *p* < 0.001, Q = 8.174, IFI = 0.976, TLI = 0.852, CFI = 0.975, RMSEA = 0.167). The sports these athletes participated in included baseball, judo, soccer, taekwondo, and hockey. Among them, judo and taekwondo athletes were designated as individual sports, and baseball, soccer, and hockey were designated as team sports and classified into two groups. Therefore, individual sports accounted for 116 (59.2%) athletes and team sports accounted for 80 (40.8%) athletes.

### 2.3. Method of Analysis

The collected data were analyzed using SPSS 24.0 (SPSS Inc., Chicago, IL, USA), SPSS PROCESS Macro, and Amos 24.0 (IBM, New York, NY, USA). The analysis involved several steps: First, a frequency analysis was conducted. Second, the reliability of each measurement tool was checked by calculating Cronbach’s alpha values, and the validity of the constructs was confirmed through confirmatory factor analysis (CFA). Third, Pearson’s product-moment correlation was calculated for the major variables. Fourth, the SPSS PROCESS Macro [50] was used to explore the moderating effect of individual and team sports on the relationship between transformational leadership, social norms, and team cohesion. Significance tests were conducted using a 95% confidence interval (C.I.) and an alpha level of 0.05. The PROCESS Macro program is an analysis tool capable of analyzing moderator, mediation, and conditional indirect effects [50]. It can analyze up to 76 models and extract direct effects, indirect effects, and specific indirect effects.

## 3. Results

### 3.1. Result of Statistical and Correlation Analyses

The descriptive statistics of study variables, including mean, standard deviation, skewness, and kurtosis are listed in Table 1. The data showed normal distribution as skewness and kurtosis fell within the recommended ranges, from −2 to +2 and from −7 to +7, respectively [51] Correlations were performed to examine overall relationships between variables, and all variables were found to be correlated below 0.78 (see Table 1). Specifically, our findings indicate that transformational leadership was positively (+) significantly correlated with social norms and team cohesion. Additionally, social norms showed a positive (+) and significant correlation with team cohesion.

### 3.2. Path Analysis of Transformational Leadership, Social Norms, and Team Cohesion

**Figure 1:**
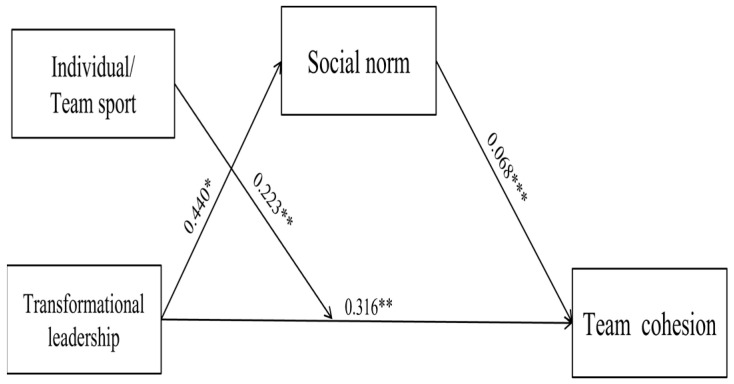
Finalized hypothesized model. Note. The values presented in this figure are unstandardized coefficients; * *p* < 0.05, ** *p* < 0. 01, *** *p* < 0.001.

The results of verifying the path analysis of transformational leadership, social norms, and team cohesion are shown in Figure 1 and Table 2. First, transformational leadership had a significant positive effect on team cohesion (B = 0.316, t = 2.799, *p* < 0.01); Hypothesis 1 was accepted. Second, transformational leadership had a significant positive effect on social norms (B = 0.440, t = 2.661, *p* < 0.001); Hypothesis 2 was accepted. Third, social norms had a significant positive effect on team cohesion (B = 0.068, t = 4.361, *p* < 0.001); Hypothesis 3 was accepted. Each path appeared meaningfully. In the next step, the moderating effect of individual/team sport athletes was tested on the relationship between transformational leadership and team cohesion.

### 3.3. Moderating Effects of Individual/Team Sports Athletes on the Relationship between Transformative Leadership and Team Cohesion

**Figure 2:**
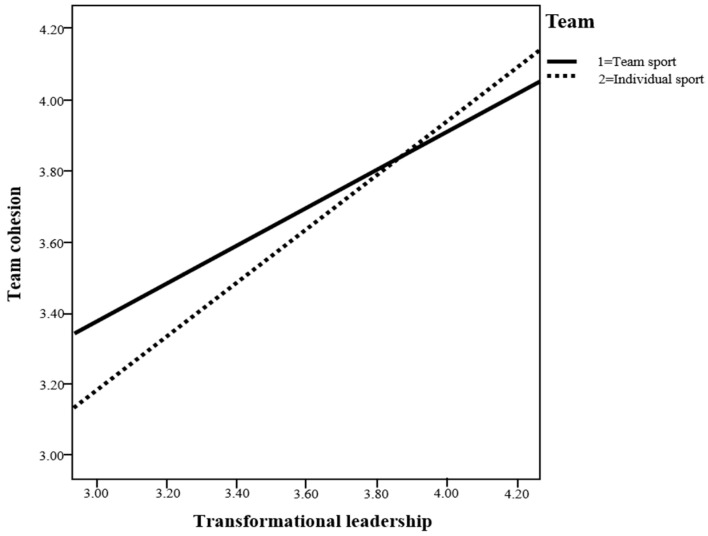
Transformational leadership and the moderating effect of individual/team sport athletes.

The moderating effect of individual/team sports athletes on the relationship between transformational leadership and team cohesion is shown in Figure 2 and Table 2. The moderating effect of transformational leadership and individual/team sports athletes was significant. For individual sports athletes, the value was 0.762 (*CI:* 0.662 to 0.861), and for team sports athletes, it was 0.539 (*CI:* 0.438 to 0.639), both of which represent a significant moderating effect (Hypothesis 4 was accepted). In particular, the value of the moderating effect was slightly higher in individual sports athletes (see Figure 2).

## 4. Discussion

Team leaders play an important role in supporting players to achieve their planned level of performance and success. For such a process, coaches must have the skills to help athletes perform difficult tasks smoothly [52]. In our study, we tried to verify the relationship between transformational leadership and team cohesion to form the team’s organizational power through social norms. We sought to investigate whether there were differential effects on team cohesion based on the perceived level of transformational leadership and whether athletes were from individual and team sports.

The results of this study show that transformational leadership has a positive effect on social norms and team cohesion and that social norms play an important role in strengthening team cohesion as a mediating variable. In particular, the interaction between transformational leadership and individual and team sports athletes was somewhat more significant for individual sports athletes. It was found that both individual and team sports players had a significant effect on team cohesion through transformational leadership. These findings have implications for improving team discipline and players’ performance in sports and have the potential to contribute to the development of sports sociopsychology based on transformational leadership theory. In light of these results, it is recommended that coaches adopt a transformative leadership role and work to establish a positive and developmental team culture. In the following, we discuss the similarities and differences of our findings with the existing literature.

First, transformational leadership was found to have a significant positive effect on team cohesion (Hypothesis 1 was accepted). It was also found that transformational leadership had a significant positive effect on team cohesion in a study of 381 college athletes by Cronin et al. [41]. Moreover, in a study of 61 female soccer players by AlTahayneh et al. [53], transformational leadership was found to enhance team cohesion. Additionally, Baird et al. [23] surveyed ice hockey players in Western Canada over an 11-week period, covering eight men’s teams and eight women’s teams. Using multilevel structural equation modeling, they analyzed athletes’ evaluations of their coaches’ transformational leadership over time. The results revealed a positive relationship between a coach’s transformational leadership and task cohesion over time.

According to Arthur et al. [54], the coach emphasizes the importance of the team mission to the players, explains the team’s vision in detail, advises players to look at difficult problems from different angles, and outlines the players’ responsibilities to the team. The authors explained that an active attitude is necessary to improve player cohesion by accommodating them. Our study also emphasizes the need for coaches to aim for transformational leadership rather than coercion to improve the team cohesion of athletes. Transformational leadership was found to have a significant positive effect on team social norms (Hypothesis 2 was accepted). Lee et al. [55] studied 401 workers in various industries in the United States and reported that transformational leadership had a positive effect on communal norms, thus supporting the results of this study.

Masi et al. [56] indirectly support the results of this study by suggesting that transformational leadership in organizations is related to organizational productivity by strengthening norms. For elite athletes in Korea, there is a high tendency to be punctual for training and to respect the hierarchy between leaders and seniors and juniors. The team reveals the need to expand the team norms to respect and consider each other. In other research, Oh [57] emphasized that the controlling coaching method, which was commonly used in the past, should be avoided, while autonomy-supporting coaching and transformational leadership should be pursued. This method can be a form of positive psychological coaching that can eliminate athletes’ exercise interruption intentions [58] and burnout [59].

Team social norms were found to have a significant positive effect on team cohesion (Hypothesis 3 was accepted). Hill et al. [47] studied 209 students, reporting that the interaction between team virtuality and norms increases team cohesion, supporting the results of this study. As a result of a study of 118 soccer players, Høigaard et al. [60] found a significant relationship between norms and cohesion. According to Forsyth [61], norms refer to common expectations for the contribution of each member of a team. Based on previous studies, our study also emphasizes the need to actively aim to increase team cohesion by increasing mutual trust between athletes, and between coaches and athletes, for a team to operate efficiently.

The interaction between transformational leadership and individual/team sports athletes was significant in both groups. However, this interaction was slightly higher in individual sports athletes. Previous studies on team athletes are compared as follows. In a study of 87 floorball players and 597 high school and college volleyball players, Wilhelmsson [62] and Kao et al. [34], respectively, found that the influence of transformational leadership and the interaction between individual and team sports athletes on team cohesion was slightly higher in individual sports players, but a significant effect on team cohesion was found in both groups. Perceptual transformational leadership appears to enhance team cohesion, supporting the results of this study. In addition, Onağ et al. [1] studied 360 team athletes in soccer, basketball, volleyball, handball, and water polo. Team cohesion, intra-team communication, and team norms led to high team satisfaction and intentions to remain on the team and exercise continuously with the team. Vella et al. [63] studied 455 youth soccer players and found that transformational leadership had a positive effect on players’ team success and beneficial developmental experiences. These studies support the results of the present study.

The outcomes of the verifications were similar in individual athletes. Park et al.’s [64] findings support the results of this study by showing that there is a significant relationship between transformational leadership and team cohesion in a study of 232 kumdo athletes. According to Oh et al. [65], transformative leadership, as perceived by 263 university taekwondo athletes, had a positive effect on pride and sports activity loyalty, indirectly supporting the findings of this study. Due to recent developments in media, sports such as track and field relay matches, mixed men’s and women’s matches, and taekwondo five-member matches, reach wider audiences through sports media. The athletes on each team show their best performance with the ability to use timing and strategy. Meanwhile, Gomes et al. [31] studied 207 swimmers (individuals) and 260 handball players (team) and confirmed the relationship between leadership and team cohesion. They found that swimmers rated coaches’ transformational leadership and team cohesion higher than handball players. These results are similar to those of the present study.

Chan et al. [66] recommended the continuous investigation of mechanisms that promote transformational leadership and team cohesion for organizational development. This study identified the mechanism of transformational leadership → social norms → team cohesion, with a significant relationship between the level of transformational leadership perceived by individual/team sports athletes and team cohesion. Our findings confirm that team cohesion is important for both individual and team athletes, and suggest that norms for team organization and consideration for teammates can strengthen team cohesion. Furthermore, this study supports the relationship between athletes’ social norms and team cohesion based on transformational leadership theory, which is expected to draw attention from researchers in the fields of sociology and ethics.

## 5. Limitations and Suggestions for Future Research

All four hypotheses of this study were accepted. However, although this study has generated meaningful research results, it is not without limitations. Athletes from five sports were divided into two groups as players of either individual or team events, which is not necessarily representative of all athletes across different sports. Future research should select research subjects by composing a population for each sport. Second, according to the study of Parazak [67], female players showed higher team cohesion than male players. Future research reveals the need to verify the relationship between transformational leadership and team cohesion according to gender. Finally, Smith et al. [4] explained that teams with high group cohesion contributed to team performance. Therefore, future studies should verify the relationship between players’ performances and team cohesion.

This study contributes to expanding the theoretical scope of transformational leadership as it was conducted in a socio-psychological context based on transformational leadership theory. To utilize the results of this study, it is recommended to create a transformative leadership role for team coaches and develop a positive team culture that promotes team social norms and systematic team work. The findings suggest that effective communication strategies and empathy between coaches and athletes are crucial.

## 6. Conclusions

The study found that transformational leadership had a significant positive effect on both social norms and team cohesion. Furthermore, social norms were shown to have a significant positive impact on team cohesion. The study also examined the interaction between transformational leadership and the type of sport (individual/team), which had a significant effect on team cohesion. Specifically, the interaction between transformational leadership and individual athletes had a stronger influence on team cohesion than the interaction between transformational leadership and team athletes.

## Figures and Tables

**Table 1:** Correlation coefficient between measurement variables.

Variables	1	2	3
Transformational leadership (1)	1.00		
Social norm (2)	0.18 **	1.00	
Team cohesion (3)	0.78 **	0.33 **	1.00
M	3.56	6.44	3.65
SD	0.81	1.89	0.68
Skewness	−0.63	−0.13	−0.54
Kurtosis	0.60	−1.10	0.52
CR	0.96	0.85	0.88
AVE	0.76	0.53	0.65
Cronbach’s α	0.95	0.94	0.82

Note. M: mean; SD: standard deviation; CR: composite reliability; AVE: average variance extracted; ** *p* < 0.01.

**Table 2:** Direct and moderating effects of transformational leadership, social norms, and team cohesion.

	B	SE	Boot S. E	t(p)	95% CI		95% BC CI	
					LLCI	ULCI	Boot LLCI	Boot ULCI
Social norm: R^2^ = 0.0352, F (1, 194) = 7.0820, *p* < 0.001								
Constant	4.879	0.604		8.074 ***	3.687	6.070		
Transformational leadership	0.440	0.165		2.661 *	0.114	0.767		
Team cohesion: R^2^ = 0.6801, F (4, 191) = 101.4962, *p* < 0.001								
Constant	2.187	0.409		5.342 ***	1.379	2.995		
Transformational leadership	0.316	0.113		2.799 **	0.093	0.538		
Social norm	0.068	0.016		4.361 ***	0.037	0.099		
Individual/Team sport	−0.865	0.257		−3.362 ***	−1.373	−0.358		
Transformational leadership * Individual/Team sport	0.223	0.071		3.149 **	0.083	0.363		
Conditional effects of the focal predictor at values of the moderator(s):								
Team sport	0.539		0.051	10.545 ***			0.438	0.639
Individual sport	0.762		0.050	15.109 ***			0.662	0.861

Note. LL, UL: bias-corrected 95% confidence interval (lower limit, upper limit); SE: standard error; * *p* < 0.05, ** *p* < 0.01, *** *p* < 0.001.

## Data Availability

The data presented in this study are available on request from the corresponding author. The data are not publicly available due to privacy issues.
